# First complete mitogenome of Massarineae and its contribution to phylogenetic implications in Pleosporales

**DOI:** 10.1038/s41598-023-49822-7

**Published:** 2023-12-17

**Authors:** Guangying Wang, Gongyou Zhang, Xiaoying lv, Yaping Wang, Yaohang Long, Xianyi Wang, Hongmei Liu

**Affiliations:** 1 Engineering Research Center of Medical Biotechnology, School of Biology and Engineering, Guizhou Medical University, Guiyang, China; 2 Engineering Research Center of Health Medicine Biotechnology of Institution of Higher Education of Guizhou Province, Guiyang, China; 3 Key Laboratory of Biology and Medical Engineering, Immune Cells and Antibody Engineering Research Center of Guizhou Province, School of Biology and Engineering, Guizhou Medical University, Guiyang, China; 4 School of Basic Medicine Science, Guizhou Medical University, Guiyang, China

**Keywords:** Ecology, Evolution, Genetics, Microbiology, Molecular biology

## Abstract

Endophytic fungi play an important role in the growth and development of traditional Chinese medicine plants. We isolated a strain of *Acrocalymma vagum* from the endophytic fungi of the traditional Chinese plants *Paris*. To accurately identify this endophytic fungal species of interest, we sequenced the mitochondrial genome of *A*. *vagum*, which is the first discovered mitochondrial genome in Massarineae. The *A*. *vagum* mitochondrial genome consists of a 35,079-bp closed circular DNA molecule containing 36 genes. Then, we compared the general sequence characteristics of *A*. *vagum* with those of Pleosporales, and the second structure of the 22 tRNAs was predicted. The phylogenetic relationship of *A*. *vagum* was constructed using two different data sets (protein-coding genes and amino acids). The phylogenetic tree shows that *A*. *vagum* is located at the root of Pleosporales. The analysis of introns shows that the number of introns increases with the increase in branch length. The results showed that monophyly was confirmed for all families in Pleosporales except for Pleosporaceae. *A*. *vagum* is an ancient species in the Pleosporales, and Pleosporaceae may require further revision. In Pleosporales, the number of introns is positively correlated with branch length, providing data for further study on the origin of introns.

## Introduction

*Acrocalymma vagum* belongs to the family Morosphaeriaceae, suborder Massarineae, and order Pleosporales^1^. The Pleosporales includes a wide range of complex organisms and is one of the largest orders of Dothideomycetes^2,3^. Most previous studies on Pleosporales relationships have focused on their morphological characteristics^4^. The different criteria employed by different fungal taxonomists to describe species result in often divergent morphological descriptions of the same fungus^5^. This has led to controversy over the taxonomic status of species in Pleosporales^6^. With the recent development of molecular sequencing technologies, molecular identification is commonly used for the identification of fungi^7^. The taxonomic status of many species in Pleosporales has been determined by molecular identification (internally transcribed spacer [*ITS*], *18S*, and *28S*)^8–10^. Differences in morphological and molecular identification result in frequent changes in the taxonomic status of many Pleosporales species^11–14^. For example, *Rhizopycnis vagum* was initially identified morphologically and then molecularly, leading to its transfer from *R*. *vagum* to *Acrocalymma*^15^.

In our previous study, we isolated an endophytic fungus from the roots of *Paris polyphylla* var. *yunnanensis*, which was morphologically and molecularly identified as belonging to *A*. *vagum*. *Paris polyphylla* var. *yunnanensis* is a valuable medicinal plant in traditional Chinese medicine. The market demand for *Paris* has increased with time, but its growth is slow and its natural reproduction rate is low. Endophytic fungi significantly promoted the growth of *Paris polyphylla* var. *yunnanensis*. Therefore, it is important to determine the distribution and characteristics of the fungal species in *Paris polyphylla* var. *yunnanensis*. In previous studies, the taxonomic status of *A*. *vagum* was generally determined by *ITS* sequencing. However, this has certain limitations due to its short molecular fragment. The mitochondrial genome is more suitable for the dating of evolutionary events, as it is believed to conform to the molecular clock hypothesis with a stable and constant mutation rate. The coding regions of the mitochondrial genome, characterized by increased length and a slower mutation rate, exhibit parallel mutations or decreased homology, which increases the reliability of phylogenetic estimates.

Owing to these advantages, mitochondrial genomes are widely used for fungal species classification. For example, Yildiz et al. identified the plant pathogenic fungus *Monilinia laxa* using a mitochondrial genome^16^; Jelen et al. used a mitochondrial genome to identify the fungus Verticillium-wilt, a plant *Verticillium nonalfalfae* pathogen^17^; and Kortsinoglou et al. used a mitochondrial genome to identify *Metarhizium*, a biocontrol agent component of entomopathogenic fungi^18^. Therefore, phylogenetic analysis of the mitochondrial genomes of *A*. *vagum* is necessary.

To date, Pleosporales has only 36 sequenced mitochondrial genomes, and there is one incomplete mitochondrial genome gene in Massarineae, with its sequence being too short to be representative. The genome’s status in the NCBI remains unverified. To fill this gap, we sequenced the complete mitochondrial genome of *A. vagum*. The complete mitochondrial genome of a species from Massarineae was obtained for annotation and phylogenetic analysis. The aim of this study was to address the following issues: (1) The general characterization of a mitochondrial genome from Massarineae; (2) Exploration of the interspecific developmental relationships among families in the order Pleosporlaes. (3) Exploration of the phylogenetic position of Massarineae in Pleosporales.

## Material and Methods

### Sample collection and DNA extraction

The strains were collected from the Chinese herbal medicine plant *Paris polyphylla* var. *yunnanensis*, located in Anshun City, Guizhou Province, China (105° 58′ 13.22″ E and 26° 02′ 22.85″ N). After screening and purification, the mycelium of *A*. *vagum* was scraped off the potato dextrose agar solid medium and ground into a powder after being frozen in liquid nitrogen. Genomic DNA was extracted using the Fungal Genome Extraction Kit (Omega Bio-tek America). Genomic DNA integrity was determined using 1% agarose gel electrophoresis. The extracted DNA was stored at − 80 °C until use.

### Mitogenome sequencing, assembly, and annotation

The whole genome of *A*. *vagum* was sequenced by Shanghai Sangon on the HiSeq 2500 platform (Illumina) with 150-bp paired-end reads. After quality evaluation and splicing, 6 GB of raw data was obtained. The sequences were assembled using Geneious Prime version 2019.2^19^. The assembled gene sequences were compared with homologous sequences retrieved from GenBank and identified through BLAST searches in the NCBI database to confirm sequence accuracy^20^. We used the MITOS^21^ web server and BLAST searches in the NCBI database (https://blast.ncbi.nlm.nih.gov/Blast.cgi) to annotate the assembled mitogenomes with mold protozoan mitochondrial genetic codes^20^ and the tRNAscan-SE 1.21 search server to identify the locations of the tRNA genes^22,23^. The *12S* and *16S* rRNA genes were identified based on the locations of the adjacent tRNA genes and compared with the sequences of other Pleosporales mitogenomes in the NCBI. ORF Finder in Geneious Prime was used to predict the protein-coding gene (PCG) locations in the mold protozoan mitochondria.

### Evolutionary rates of the mitochondrial genes

The synonymous sites (Ks), nonsynonymous substitution sites (Ka), and their ratios (Ka/Ks) are often employed to measure evolutionary rates^24^. Therefore, we chose 13 PCGs (*atp6*, *cob*, *cox1*, *cox2*, *cox3*, *nad1*, *nad2*, *nad3*, *nad4*, *nad4l*, *nad5*, *rps3*, and *nad6*) of 18 Pleosporales to calculate the values of Ka, Ks, and Ka/Ks. These genes were aligned using MEGA 7.0^25^ according to the codons (parameters: Gap opening penalty: 400; Gap extension penalty: 0.2; and Delay divergent cutoff: 30%), and the Ka/Ks ratio was calculated using DnaSP ver. 5^26^.

### Mitogenome annotation and sequence analysis

Mapping and comparative analysis of the mitochondrial genomes were performed on CGview (https://proksee.ca/). The codon usage frequencies of amino acids in the mitochondrial genome were analyzed using CodonW and MEGA 7.0^25^. The relative synonymous codon usage value and codon usage frequency in the mitochondrial genome were analyzed with MEGA 7.0^25^. Finally, the chain asymmetry was calculated using the following formula^27^:

$${\text{AT skew}} = \left( {{\text{A }} - {\text{ T}}} \right)/\left( {{\text{A }} + {\text{ T}}} \right){\text{ and}}\;{\text{GC}}\;{\text{skew}} = \left( {{\text{G }} - {\text{ C}}} \right)/\left( {{\text{G }} + {\text{ C}}} \right)$$

### Phylogenetic analysis

Eighteen Pleosporales [Astrosphaeriellaceae (1), Corynesporascaceae (1), Coniothyriaceae (1), Didymellaceae (3), Morosphaeriaceae (1), Phaeosphaeriaceae (1), Pleosporaceae (9), and Shiraiaceae (1)]^28–42^ were selected to construct the phylogenetic tree after the removal of unverified sequences, those that lacked an accurate scientific name, or were repetitive. Due to the loss of the rsp3 gene in some species during evolution, only 12 PCGs were used to construct the phylogenetic tree. Phylogenetic analysis was performed using the alignments of the 12 PCGs of complete or near-complete mitogenomes of the Pleosporales species. The three species of *Zymoseptoria tritici*, *Pseudocercospora fijiensis*, and *Zasmidium cellare* were used as outgroups^43–45^ (Table S1).

The 12 PCGs were aligned using the TranslatorX online tool, employing MAFFT to perform the protein alignment^34,46–48^. The 12 resulting alignments were assessed and manually corrected using MEGA version 7.0 program^25^. For the phylogenetic analyses, the maximum likelihood (ML) and Bayesian inference (BI) methods were employed to construct the ML and BI trees based on two datasets (PCG, the 12 PCGs; AA and amino acid sequences of the 12 PCGs). ML analysis was performed with 1000 rapid bootstrapping replicates using iqtree^49^, whereas the BI analysis was performed in MrBayes 3.2.7a with four chains and sampling of the chains every 1000 generations^50^. Two independent runs of 10 million generations were performed. After the average standard deviation of the split frequencies decreased to < 0.001, the initial 25% of the samples were discarded as the burn-in and the remaining trees were used to generate a consensus tree and calculate the posterior probabilities^51^. The BI and ML analyses were performed on the CIPRES Science Gateway (https://www.phylo.org) website, and the phylogenetic trees were visualized using FigTree 1.4.2^52^.

### Ethical approval

This article does not contain any studies with human participants performed by any of the authors.

## Results

### Genome organization

**Figure 1:**
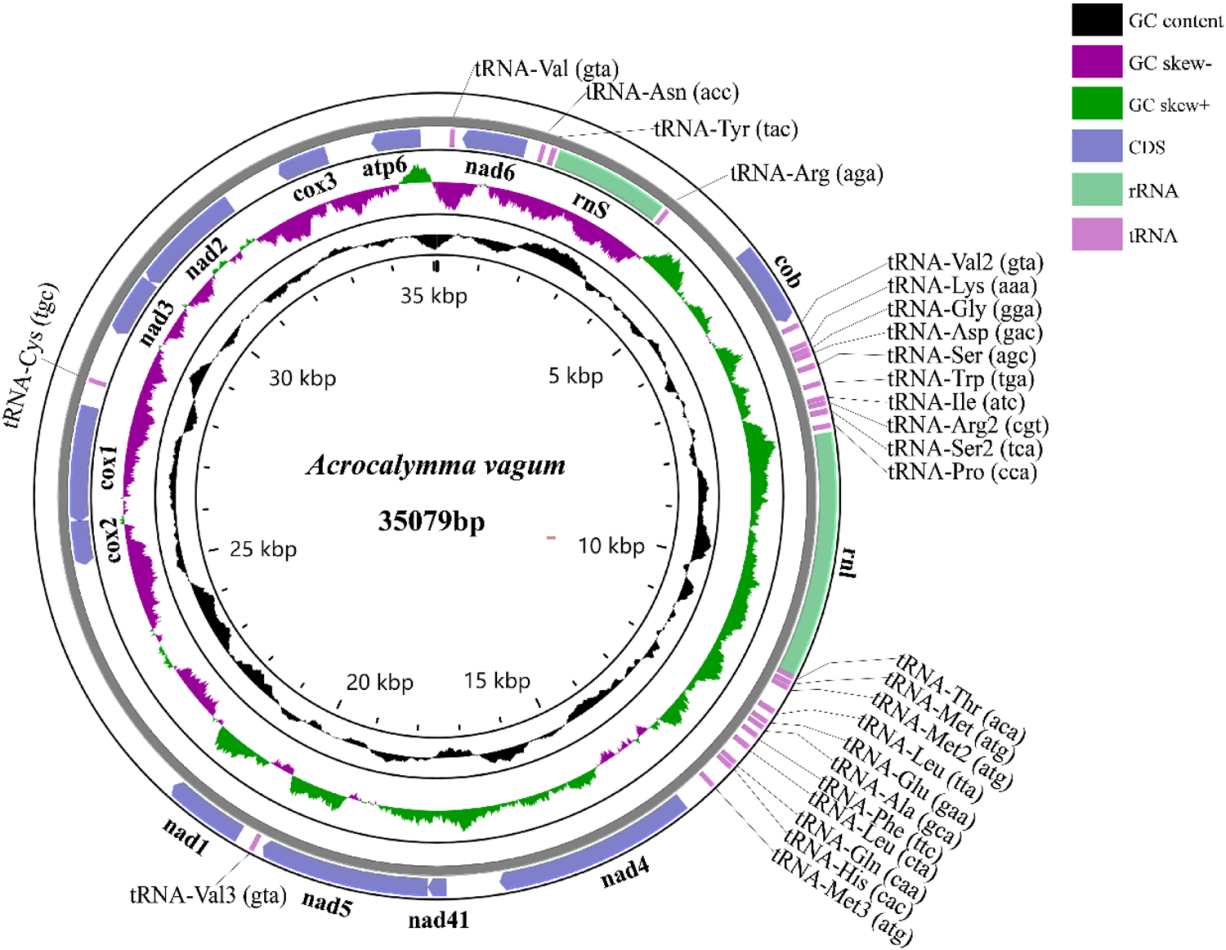
Circular map of the *Acrocalymma vagum* mitochondrial genome.

The mitochondrial genome of the fungal strain is a closed circular DNA molecule with a length of 35,079 bp. It contains 36 genes, including 12 PCGs, 2 rRNA genes, and 22 tRNA genes (Fig. 1). The 12 PCGs ranged in length from 270 bp (*nad4l*) to 2886 bp (*nad4*). The AT content of these 12 PCGs ranged from 67.3 to 78.2%, which was consistent with the previously reported genomes of mitotic Pleosporales species^28,42^. The AT skew of these 12 PCGs was − 0.167–0.167, and the GC skew was − 0.089–0.205. The 12 PCGs contained a subunit in the F0 region of the ATP synthase complex (*atp6*), three cytochrome oxidases (*cox1*, *cox2*, and *cox3*), seven subunits of the electron transport chain complex I (*nad1*, *nad2*, *nad3*, *nad4*, *nad4l*, *nad5*, and *nad6*), and a subunit (*cob*) of complex III. The strand where most genes are located is referred to as the J-strand (majority strand), and the strand where few genes are located is referred to as the N-strand (minority strand). It has five PCGs, which are encoded by the N-strand (*cob*, *nad1*, *nad4*, *nad4l*, and *nad5*), and the remaining seven PCGs are encoded by the J-strand (*atp6*, *nad2*, *nad3*, *nad6*, *cox1*, *cox2*, *and cox3*) (Table S2).

### PCGs with codon usage and nucleotide composition

**Figure 2:**
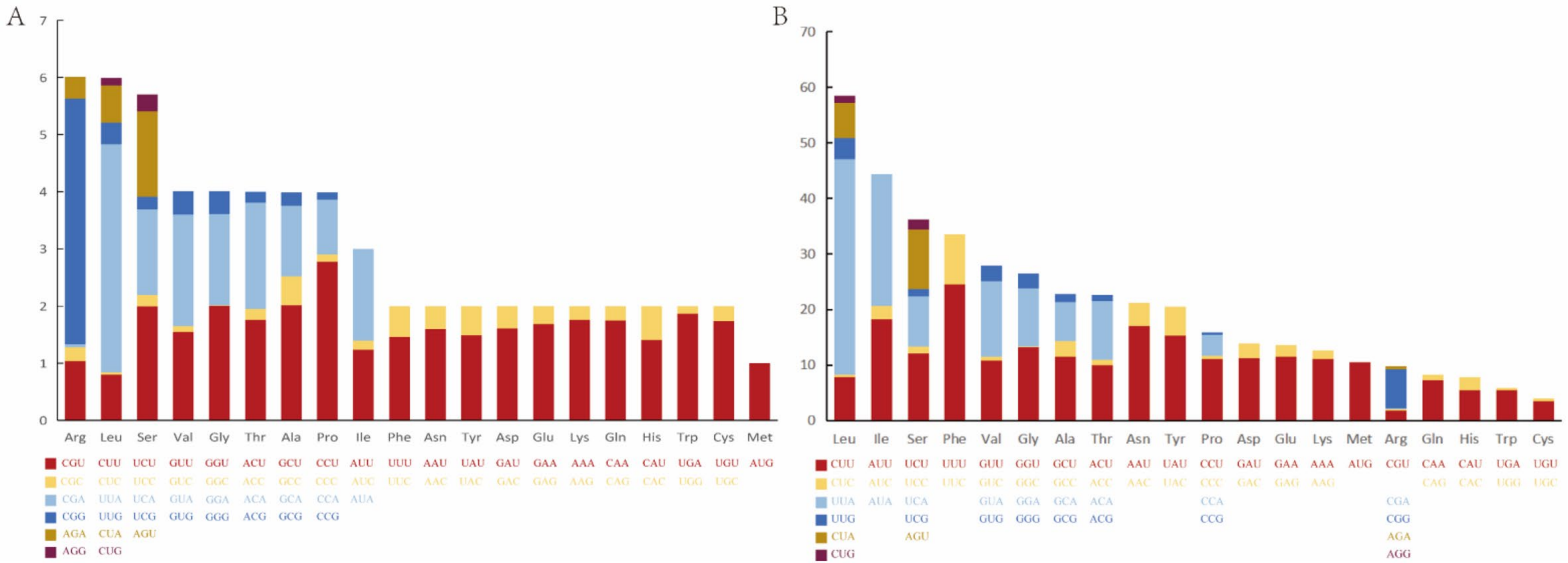
Basic characteristics of codons in the *Acrocalymma vagum* mitochondrial genome. (**A**) Codon distribution in the *Acrocalymma vagum* mitogenome. Numbers on the y-axis refer to the total number of codons. Codon families are shown on the x-axis. (**B**) Codon usage in the mitochondrial genome in *Acrocalymma vagum*. Different colored boxes represent codon usage.

To understand the bias in codon usage of the mitchondrial genes in *A*. *vagum*, the codon usage in the PCGs was determined. A statistical analysis of codon usage in the PCGs region of *A*. *vagum* was conducted (Fig. 2A). Among the *A*. *vagum*, the most frequently used codon was UUA (for leucine; Leu) and the most frequently used amino acids were 58.5 leucine (Leu), 44.4 isoleucine (Ile), 36.2 serine (Ser), and 33.5 phenylalanine (Phe). In addition, CGC, GGC, CGG, CUC, and CGG were the five codons with the lowest usage rates (Fig. 2B).

**Figure 3:**
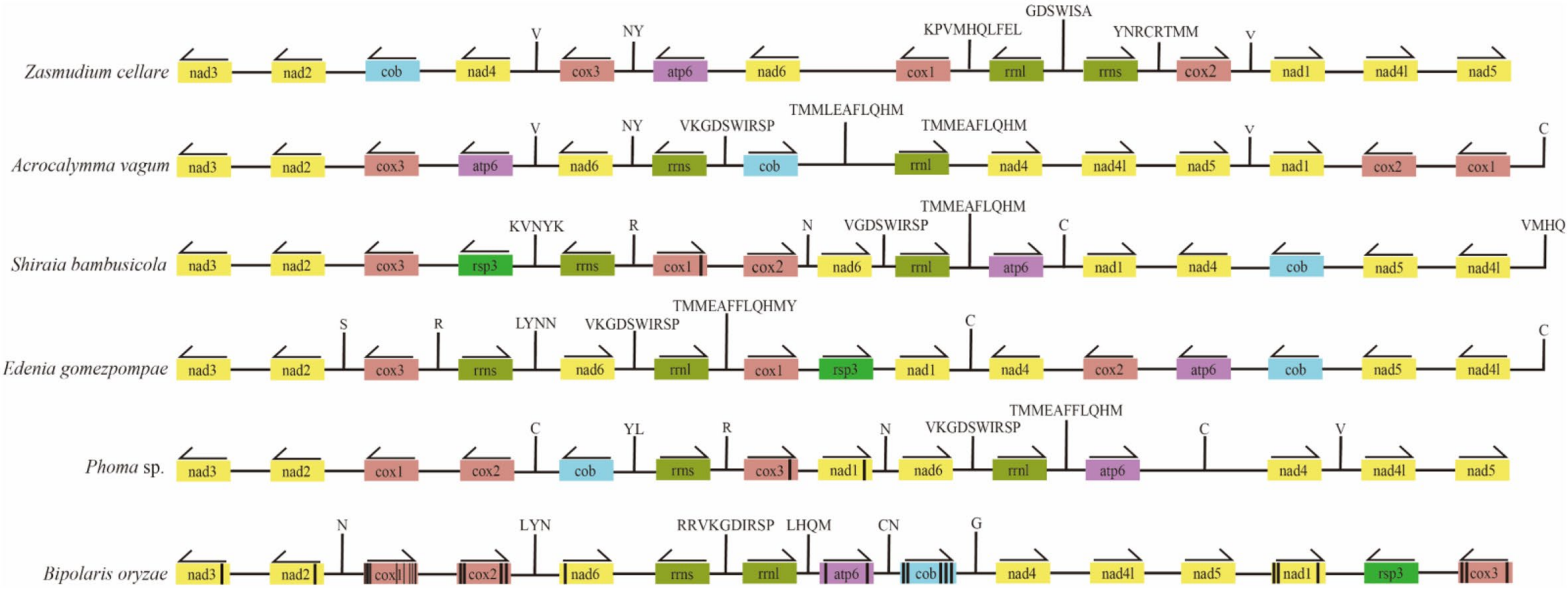
The order of the species in this figure, from top to bottom, is determined by their relatedness to *Acrocalymma vagum*, ranging from closely related to distant. Additionally, positioned at the top are the outgroup species *Z*. *cellare*. Comparative analysis of the nucleotide sequences for each protein-coding gene and two ribosomal DNA genes among *A*. *vagum*, *S*. *bambusicola*, *E*. *gomezpompae*, *Phoma* sp., and *B*. *oryzae*. The same genes are marked with the same color, and the black color in a gene represents an intron in that gene.

To understand the differences in mitochondrial genome composition between two species, the mitochondrial genomes of *A*. *vagum* and outgroup species (*Z*. *cellare* and *A*. *vagum* relatives from close to distant *Shiraia bambusicola*, *Edenia gomezpompae*, *Phoma* sp., and *Bipolaris oryzae*) were linearized and plotted (Fig. 3). The figure shows that both *A*. *vagum* and *Phoma* sp. lack the *rsp3* gene. Therefore, we further performed the evolutionary analysis of *rsp3*.

### Evolutionary rates of mitochondrial genes

**Figure 4:**
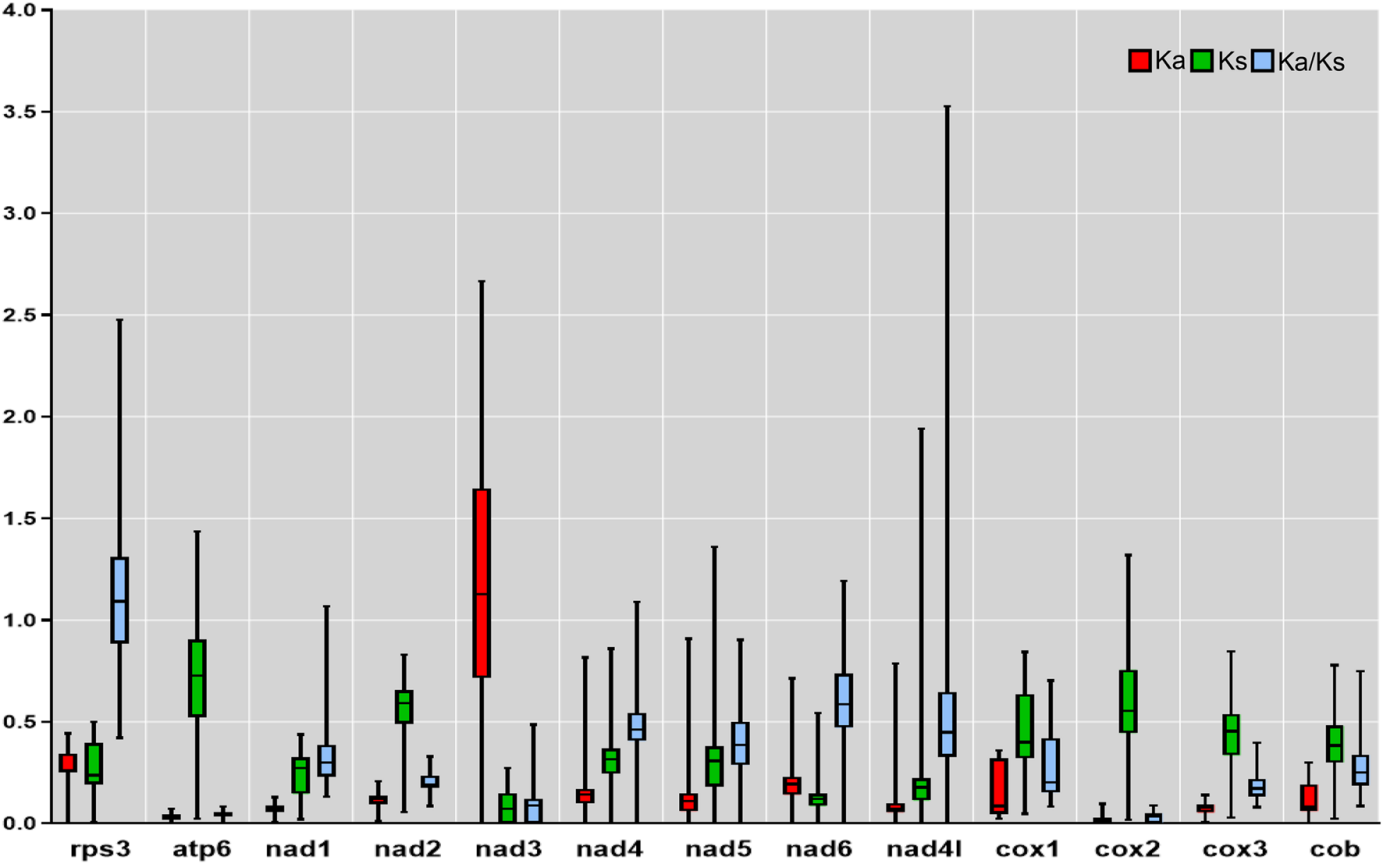
The Ka (nonsynonymous) and Ks (synonymous) values, as well as their Ka/Ks ratios appear as red, green, and blue box plots, respectively. The bar represents the standard deviation, median, and the values between the first and third quartiles of Ka, Ks, and Ka/Ks.

To detect the nature of the evolutionary selection pressure in *A*. *vagum*, we selected 13 mitochondrial genes to calculate Ka, Ks, and Ka/Ks values. The results (Fig. 4) showed that the Ka/Ks ratio ranged from 0.06 to 1.02 for the mitochondrial genes, suggesting that these genes underwent negative selection pressure in the evolution process. The mean Ka/Ks ratio of these mitochondrial genes was 0.407. The mitochondrial genes with the highest Ka/Ks ratio were *rps3* (1.02), *nad6* (0.69), and *nad4* (0.62), with *rps3* undergoing positive selection. This may explain the phenomenon of the rps3 gene absence in *A. vagum*, while other genes underwent purifying selection during their evolutionary process.

### tRNAs and rRNAs

**Figure 5:**
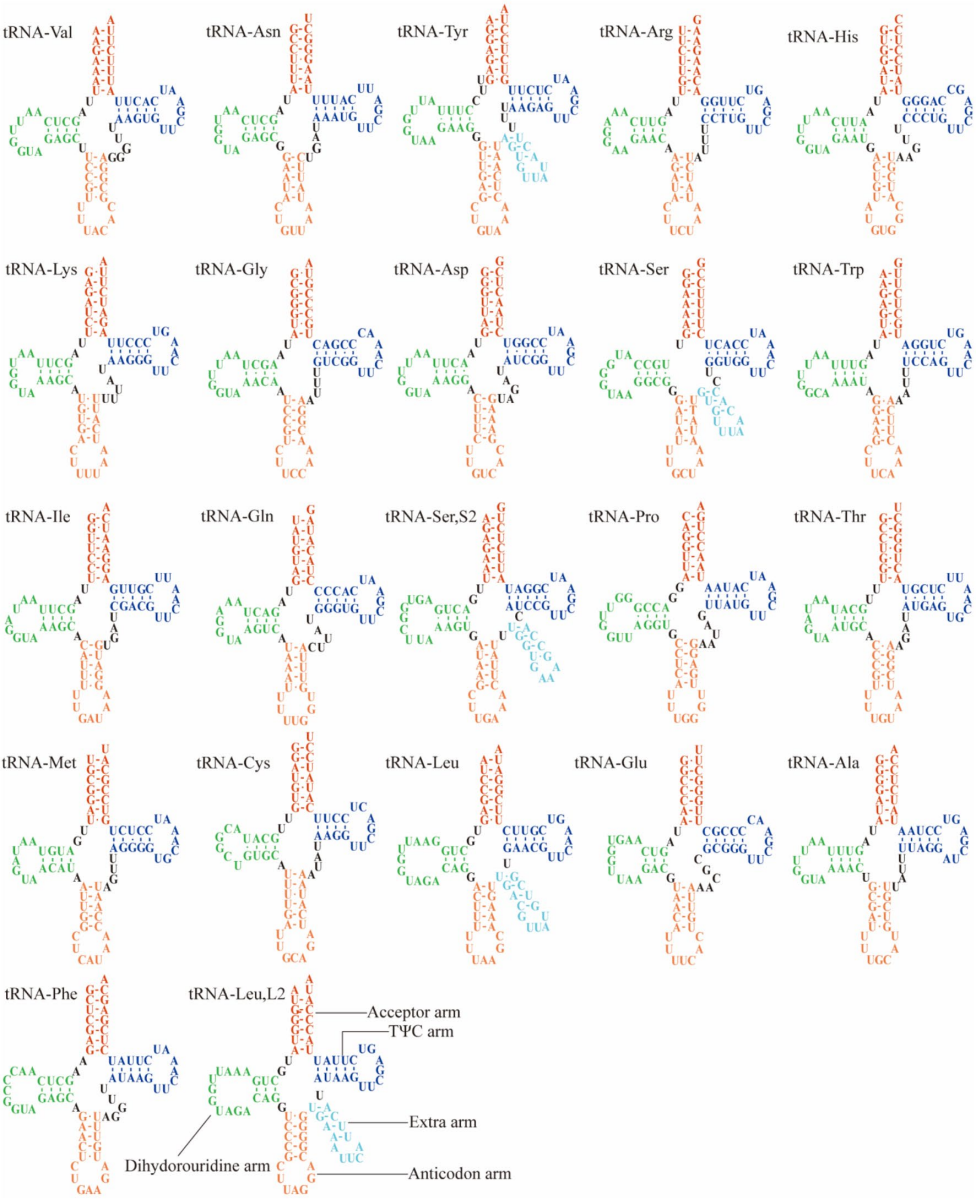
Predicted secondary structures of the 22 tRNAs of the *Acrocalymma vagum* mitochondrial genome.

Twenty-two tRNA genes were located in the *A*. *vagum* mitochondrial genome, Arg, Ser, and Leu were encoded by two tRNA genes with different anticodons; Val and Met were encoded by three tRNA genes with the same anticodon; and the tRNA arms of the same AA codon were different. These tRNAs have a typical highly conserved clover secondary structure (Fig. 5), and 55 G-U mismatches were found, most of which were distributed around *rrnL*. These tRNAs ranged in length from 71 to 85 bp, with a total length of 2003 bp, representing 5.71% of the mitochondrial genome length. The AT content of the tRNAs was 60.2%, displaying an AT skew of − 0.167–0.190 and a GC skew of − 0.22–0.375. The mitochondrial genome of *A*. *vagum*, as with other eukaryotes, had two rRNA genes (*16S* and *12S*), namely the small subunit rRNA (*rrnS*) and large subunit rRNA (*rrnL*). The lengths of the *rrnL* and *rrnS* genes were 1792 and 3493 bp, respectively.

### Phylogenetic relationships

**Figure 6:**
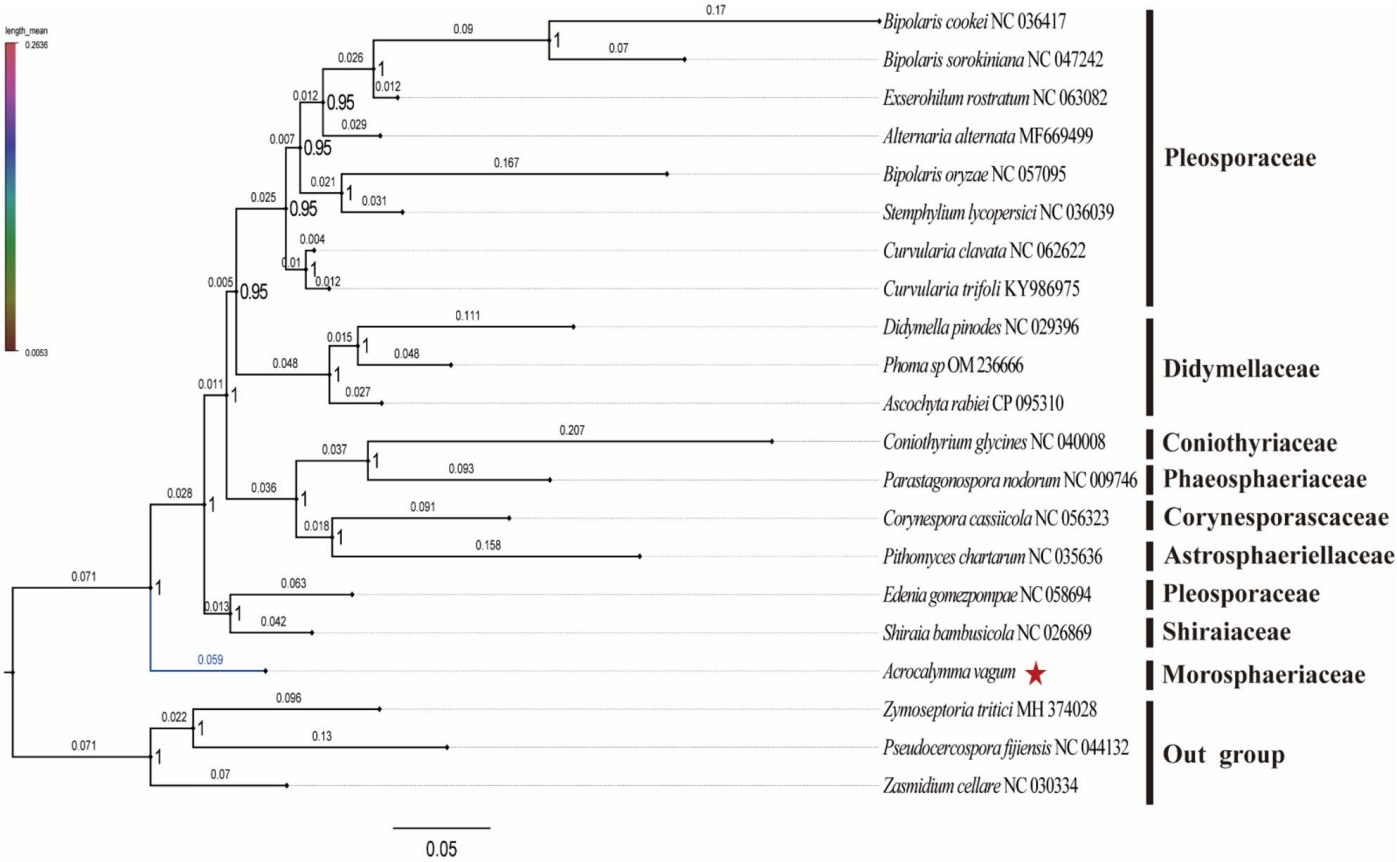
Phylogenetic relationships of Pleosporales inferred by MrBayes 3.2.6 based on the nucleotides of the first and second codons of the 12 protein-coding genes (11,688 bp) and two rRNAs. Numbers on nodes are the percentage frequency with which a cluster appears in a bootstrap test of 1000 runs (≥ 95%). The number on the branch represents the length of the branch.

To obtain more evidence to conduct the classification and understand the evolutionary history of the mitochondrial genome, we used the identical and well-supported tree topology based on two datasets, PCG and AA, using BI and ML methods. The branching nodes of all trees showed a high degree of node support (bootstrap support BS > 90) and BI (posterior probabilities PP = 1.00). In the four phylogenetic trees of BI-AA, ML-AA, BI-PCG, and ML-PCG, *A*. *vagum* has the smallest evolutionary distance. The main difference between the AA and PCG analyses was that the species closest to *A*. *vagum* in the phylogenetic relationship were *Ascochyta rabiei* and *S*. *bambusicola*, respectively (Fig. 6, Figs. S1–S3). The BI-PCG and ML-PCG analyses align more closely with previous phylogenetic studies^41,53^.

At the family level, the results of phylogenetic relationships indicate the well-verified monophyletic groups of Astrosphaeriellaceae, Orynesporascaceae, Coniothyriaceae, Didymellaceae, Morosphaeriaceae, Phaeosphaeriaceae, and Shiraiaceae, while the monophyletic group of Pleosporaceae is questioned. At the genus level, the monophylies of all genera, except for *Bipolaris*, were well verified in Pleosporaceae, and *B*. *oryzae* was distantly related to the other two species.

Our results (Fig. 6) show that *A*. *vagum* belongs to the family Morosphaeriaceae and is located at the base of the phylogenetic tree of Pleosporales, which is an ancient species under the Pleosporales. Morosphaeriaceae is most closely related to Shiraiaceae based on phylogenetic distance, exhibiting a greater genetic distance from species within Pleosporaceae. The results showed that the combined mitochondrial gene set was suitable as a reliable molecular marker for the analysis of the phylogenetic relationships among the Pleosporales species.

## Discussion

The basic information regarding the mitochondrial genome reveals a significantly higher AT than GC content in *A*. *vagum*, which is attributed to the fact that the four most frequently used codons (UUA, AUA, UUU, and AAU) in the mitochondrial genome of *A*. *vagum* are all composed of A and T. This is the same reason for the high AT content in Pleosporales in general^42,54^. Mitochondrial genome length varied from 30,836 bp (*Phoma* sp.) to 13,600 bp (*B*. *sorokiniana*) in 18 Pleosporales. There is a large gap in the mitochondrial genome size among species, and this is related to the number of introns. As the number of introns increases, so does the length of the genome, which is supported by previous studies on fungi^55,56^. It was observed that the number of introns increased with the evolutionary distance. In the outer group species *Zasmudium cellare* and the species with the smallest evolutionary distance *A*. *vagum*, no introns were present. Among the three species closely related to *A*. *vagum*, namely *S*. *bambusicola*,* E*. *gomezpompae*, and *Phoma* sp., the number of introns was 1, 0, and 2, respectively. The phylogenetic results showed that the number of introns increased with the evolutionary distance of the species; e.g., species in the clade containing *B*. *oryzae* have higher numbers of introns than species more closely related to* A*. *vagum*. There are two generally accepted theories regarding the origin of introns. The first theory suggests that introns were abundant in the ancestral mitochondrial genome but were subsequently lost in most lineages^57,58^. The second theory supports intron mobility and expansion within genes due to events of horizontal transfer, even between distant phylogenetically related species^59,60^. The trend of the number of introns gradually increasing with evolutionary distance is consistent with the second conjecture. With the gradual evolution of the species, the horizontal and vertical transfer of genes in the mitochondrial genome leads to an increased number of introns.

The results of the phylogenetic relationships show that Pleosporaceaeas is a paraphyletic group. Among them, *E*. *gomezpompae* is genetically distant from other species of Pleosporaceae but closely related to Shiraiaceae. This result differs from the report by Cui et al., who proposed placing *Edenia* in Pleosporaceae based on the phylogenetic analysis of *ITS* rDNA^61^. However, our results are consistent with the phylogenetic tree of the mitochondrial genome reported by Huang et al.^41^. There are two reasons for this taxonomic difference: (1) There are limitations in constructing phylogenetic trees from *ITS* rDNA sequences alone, and species closer to *Edenia* may not have been included in the phylogenetic tree construction. (2) Morphological characteristics were not compared between species in the family Pleosporaceae. The phylogenetic tree constructed in this study included more species of the Pleosporales order. Therefore, we suggest that *Edenia* be merged into Shiraiaceae after being removed from Pleosporaceae. To further confirm the relationship between the genus *Edenia* and the family Shiraiaceae, more morphological features of other species of *Edenia* and Shiraiaceae need to be compared. We observed that *B*. *cooke* and *B*. *sorokiniana* in the genus *Bipolaris* are in the same clade. Another species in the genus *Bipolaris*, *B*. *oryzae*, was more closely related to the genus Stemphylium. Therefore, we question the monophyly of *Bipolaris*. However, establishing the relationship between *B*. *oryzae* and Stemphylium requires additional morphological and molecular data to prove this relationship.

Line plots of the mitochondrial genome show that although the PCGs and rRNAs were arranged in vastly different orders in the mitochondria, they still showed considerable homology. Our results support the widespread distribution of the *nad2*–*nad3*, *nad4l*–*nad5*, and *cox1*–*cox2* pairs in Pleosporlaes, and tRNA was mostly distributed on both sides of the *rrnl*^42^. *A*. *vagum* and the other four strains showed significant differences in the mitochondrial gene arrangement. However, there were four genes in the same position, maintaining a certain degree of homology. This indicates that numerous rearrangement events occurred in *A*. *vagum* and its close relatives during its evolution. The mitochondrial gene arrangements of *S*. *bambusicola* and *E*. *gomezpompae* are highly similar. This provides further evidence that *Edenia* should be incorporated into the family Shiraiaceae.

## Conclusion

In this study, we sequenced and annotated the complete genome of *A*. *vagum*, an endophytic fungus of the highly valued Chinese herbal medicine *Paris polyphylla* var*. yunnanensis*. Then, the mitochondrial genome sequence was uploaded to NCBI (GenBank accession number: OQ509476). The mitochondrial characteristics (gene content, size, and order, base composition, PCG codon usage, and tRNA secondary structure) were placed into the order Pleosporlaes for their comparison. It was proven that *A*. *vagum* belongs to the family Massarineae and is an ancient species of the order Pleosporales. This study is the first to report the complete mitochondrial genome obtained from the family Massarineae. In the mitochondrial genome signature, it was found that the mitochondrial introns gradually increased with species evolution. Mitochondria within Massarineae and closely related species have a lower intron count than distant relatives, as observed in the species *Bipolaris*. In addition, we performed a phylogenetic analysis of Pleosporlaes, which was used to determine the phylogenetic relationships between the species in Pleosporlaes. Based on the phylogenetic tree, *Edenia* should be placed in the family Shiraiaceae, and Morosphaeriaceae and Shiraiaceae are sister groups with high homology ratings. This study not only improved our phylogenetic understanding of Pleosporlaes but also provided a new method to identify the distribution of endophytic fungi in Chinese herbal medicine.

### Relevant legislations, permitting and consent

The collection of plant material must comply with relevant institutional, national, and international guidelines and legislation as well as IUCN Policy Statement on Research Involving Species at Risk of Extinction and Convention on the Trade in Endangered Species of Wild Fauna and Flora.

### Supplementary Information

Supplementary Figures.

Supplementary Table S1.

Supplementary Table S2.

## Data Availability

The data that support the findings of this study will be available in GenBank at https://www.ncbi.nlm.nih.gov/, with accession number OQ509476.

## References

[1] Crous PW (2014). Fungal Planet description sheets: 214–280. Persoonia.

[2] Zhang Y (2009). Multi-locus phylogeny of Pleosporales: A taxonomic, ecological and evolutionary re-evaluation. Stud. Mycol..

[3] Schoch CL (2009). A class-wide phylogenetic assessment of Dothideomycetes. Stud. Mycol..

[4] Zhang Y, Crous PW, Schoch CL, Hyde KD (2012). Pleosporales. Fungal Divers.

[5] Xu J (2016). Fungal DNA barcoding. Genome.

[6] Mugambi GK, Huhndorf SM (2009). Molecular phylogenetics of Pleosporales: Melanommataceae and Lophiostomataceae re-circumscribed (Pleosporomycetidae, Dothideomycetes, Ascomycota). Stud. Mycol..

[7] Guo LD (2003). Molecular identification of white morphotype strains of endophytic fungi from *Pinus tabulaeformis*. Mycol. Res..

[8] Teimoori-Boghsani Y (2019). Endophytic fungi of native salvia abrotanoides plants reveal high taxonomic diversity and unique profiles of secondary metabolites. Front. Microbiol..

[9] Ameen F, Stephenson SL, AlNadhari S, Yassin MA (2021). Isolation, identification and bioactivity analysis of an endophytic fungus isolated from Aloe vera collected from Asir desert, Saudi Arabia. Bioprocess Biosyst. Eng..

[10] Xiao JL (2021). Isolation and screening of stress-resistant endophytic fungus strains from wild and cultivated soybeans in cold region of China. Appl. Microbiol. Biotechnol..

[11] Manamgoda DS (2014). The genus *Bipolaris*. Stud. Mycol..

[12] Tanaka K (2015). Revision of the Massarineae (Pleosporales, Dothideomycetes). Stud. Mycol..

[13] Ahmed SA (2014). Revision of agents of black-grain eumycetoma in the order Pleosporales. Persoonia.

[14] Wanasinghe DN (2017). Phylogenetic revision of *Camarosporium* (Pleosporineae, Dothideomycetes) and allied genera. Stud. Mycol..

[15] Trakunyingcharoen T (2014). Mycoparasitic species of *Sphaerellopsis*, and allied lichenicolous and other genera. IMA Fungus.

[16] Yildiz G, Ozkilinc H (2020). First characterization of the complete mitochondrial genome of fungal plant-pathogen Monilinia laxa which represents the mobile intron rich structure. Sci. Rep..

[17] Jelen V, de Jonge R, Van de Peer Y, Javornik B, Jakše J (2016). Complete mitochondrial genome of the Verticillium-wilt causing plant pathogen *Verticillium nonalfalfae*. PLoS One.

[18] Kortsinoglou AM, Saud Z, Eastwood DC, Butt TM, Kouvelis VN (2020). The mitochondrial genome contribution to the phylogeny and identification of Metarhizium species and strains. Fungal Biol..

[19] Kearse M (2012). Geneious Basic: An integrated and extendable desktop software platform for the organization and analysis of sequence data. Bioinformatics.

[20] Altschul SF (1997). Gapped BLAST and PSI-BLAST: A new generation of protein database search programs. Nucleic Acids Res,.

[21] Bernt M (2013). MITOS: Improved de novo metazoan mitochondrial genome annotation. Mol. Phylogenet. Evol..

[22] Laslett D, Canbäck B (2008). ARWEN: A program to detect tRNA genes in metazoan mitochondrial nucleotide sequences. Bioinformatics.

[23] Schattner P, Brooks AN, Lowe TM (2005). The tRNAscan-SE, snoscan and snoGPS web servers for the detection of tRNAs and snoRNAs. Nucleic Acids Res..

[24] Kryazhimskiy S, Plotkin JB (2008). The population genetics of dN/dS. PLoS Genet..

[25] Kumar S, Stecher G, Tamura K (2016). MEGA7: Molecular evolutionary genetics analysis version 7.0 for bigger datasets. Mol. Biol. Evol..

[26] Librado P, Rozas J (2009). DnaSP v5: A software for comprehensive analysis of DNA polymorphism data. Bioinformatics.

[27] Perna NT, Kocher TD (1995). Patterns of nucleotide composition at fourfold degenerate sites of animal mitochondrial genomes. J. Mol. Evol..

[28] Zaccaron AZ, Bluhm BH (2017). The genome sequence of *Bipolaris cookei* reveals mechanisms of pathogenesis underlying target leaf spot of sorghum. Sci. Rep..

[29] Song N, Geng Y, Li X (2020). The mitochondrial genome of the phytopathogenic fungus *Bipolaris sorokiniana* and the utility of mitochondrial genome to infer phylogeny of Dothideomycetes. Front. Microbiol..

[30] Ma Q (2022). Comparative mitochondrial genome analyses reveal conserved gene arrangement but massive expansion/contraction in two closely related *Exserohilum pathogens*. Comput. Struct. Biotechnol. J..

[31] Liao M, Chen C, Li Q (2017). The complete mitochondrial genome of *Alternaria alternata* (Hypocreales: Nectriaceae). Mitochondrial. DNA B Resour..

[32] Franco MEE (2017). The mitochondrial genome of the plant-pathogenic fungus *Stemphylium lycopersici* uncovers a dynamic structure due to repetitive and mobile elements. PLoS One.

[33] Yuan XL (2020). Characterization of nuclear and mitochondrial genomes of two tobacco endophytic fungi *Leptosphaerulina chartarum* and *Curvularia trifolii* and their contributions to phylogenetic implications in the Pleosporales. Int J Mol Sci.

[34] Deng G (2019). The complete mitochondrial genome of *Cochliobolus miyabeanus* (Dothideomycetes, Pleosporaceae) causing brown spot disease of rice. Mitochondrial. DNA B Resour..

[35] Chen C (2019). Characterization of the complete mitochondrial genome of *Corynespora cassiicola* (Pleosporales: Dothideomycetes), with its phylogenetic analysis. Mitochondrial. DNA B Resour..

[36] Shah RM (2020). Reference Genome Assembly for Australian *Ascochyta rabiei* Isolate ArME14. G3.

[37] Panteleev S, Mozharovskaya L, Kiryanov P, Kagan D, Baranov OY (2022). Structural and functional organisation of the phytopathogenic fungi Phoma sp. 1 mitochondrial genome. Proc. Natl. Acad. Sci. Belarus Biol. Ser..

[38] Hane JK (2007). Dothideomycete plant interactions illuminated by genome sequencing and EST analysis of the wheat pathogen *Stagonospora nodorum*. Plant Cell.

[39] Stone CL (2018). Annotation and analysis of the mitochondrial genome of *Coniothyrium glycines*, causal agent of red leaf blotch of soybean, reveals an abundance of homing endonucleases. PLoS One.

[40] Wu D, Zhou L, Xue J, Xia Q, Meng L (2022). Characterization of two new *Apodemus mitogenomes* (Rodentia: Muridae) and mitochondrial phylogeny of Muridae. Diversity.

[41] Huang L, Mu Y, Zhang X, Chang K, Zhang J (2022). The mitochondrial genome of the endophyte *Edenia gomezpompae* CRI Eg3 isolated from sweet potato. Mitochondrial. DNA B Resour..

[42] Shen XY (2015). Characterization and phylogenetic analysis of the mitochondrial genome of *Shiraia bambusicola* reveals special features in the order of pleosporales. PLoS One.

[43] Torriani SF, Brunner PC, McDonald BA (2011). Evolutionary history of the mitochondrial genome in *Mycosphaerella* populations infecting bread wheat, durum wheat and wild grasses. Mol. Phylogenet. Evol..

[44] Arcila-Galvis JE, Arango RE, Torres-Bonilla JM, Arias T (2021). The mitochondrial genome of a plant fungal pathogen *Pseudocercospora fijiensis* (Mycosphaerellaceae), comparative analysis and diversification times of the sigatoka disease complex using fossil calibrated phylogenies. Life.

[45] Goodwin SB (2016). The mitochondrial genome of the ethanol-metabolizing, wine cellar mold Zasmidium cellare is the smallest for a filamentous ascomycete. Fungal Biol..

[46] Abascal F, Zardoya R, Telford MJ (2010). TranslatorX: Multiple alignment of nucleotide sequences guided by amino acid translations. Nucleic Acids Res..

[47] Castresana J (2000). Selection of conserved blocks from multiple alignments for their use in phylogenetic analysis. Mol. Biol. Evol..

[48] Katoh K, Rozewicki J, Yamada KD (2019). MAFFT online service: Multiple sequence alignment, interactive sequence choice and visualization. Brief Bioinform..

[49] Ronquist F (2012). MrBayes 3.2: Efficient Bayesian phylogenetic inference and model choice across a large model space. Syst. Biol..

[50] Nguyen LT, Schmidt HA, von Haeseler A, Minh BQ (2015). IQ-TREE: A fast and effective stochastic algorithm for estimating maximum-likelihood phylogenies. Mol Biol Evol.

[51] Du Y, Zhang C, Dietrich CH, Zhang Y, Dai W (2017). Characterization of the complete mitochondrial genomes of *Maiestas dorsalis* and *Japananus hyalinus* (Hemiptera: Cicadellidae) and comparison with other Membracoidea. Sci. Rep..

[52] Nobile PM (2017). Identification, classification and transcriptional profiles of dirigent domain-containing proteins in sugarcane. Mol. Genet. Genom..

[53] Tao L (2021). Plant growth-promoting activities of bacterial endophytes isolated from the medicinal plant Pairs *polyphylla* var. *yunnanensis*. World J. Microbiol. Biotechnol..

[54] Oh J, Kong WS, Sung GH (2015). Complete mitochondrial genome of the entomopathogenic fungus *Beauveria pseudobassiana* (Ascomycota, Cordycipitaceae). Mitochondrial DNA.

[55] Torriani SF (2014). Comparative analysis of mitochondrial genomes from closely related *Rhynchosporium* species reveals extensive intron invasion. Fungal Genet. Biol..

[56] Li Q (2020). The complete mitochondrial genomes of two model ectomycorrhizal fungi (Laccaria): Features, intron dynamics and phylogenetic implications. Int. J. Biol. Macromol..

[57] Goddard MR, Burt A (1999). Recurrent invasion and extinction of a selfish gene. Proc. Natl. Acad. Sci. U. S. A..

[58] Gonzalez P, Barroso G, Labarère J (1999). Molecular gene organisation and secondary structure of the mitochondrial large subunit ribosomal RNA from the cultivated Basidiomycota *Agrocybe aegerita*: A 13 kb gene possessing six unusual nucleotide extensions and eight introns. Nucleic Acids Res..

[59] Vaughn JC, Mason MT, Sper-Whitis GL, Kuhlman P, Palmer JD (1995). Fungal origin by horizontal transfer of a plant mitochondrial group I intron in the chimeric CoxI gene of Peperomia. J. Mol. Evol..

[60] Gonzalez P, Barroso G, Labarère J (1998). Molecular analysis of the split cox1 gene from the Basidiomycota *Agrocybe aegerita*: Relationship of its introns with homologous Ascomycota introns and divergence levels from common ancestral copies. Gene.

[61] Cui Y (2013). Characterization of *Edenia gomezpompae* isolated from a patient with keratitis. Mycopathologia.

