# Supplementary material for: First complete mitogenome of Massarineae and its contribution to phylogenetic implications in Pleosporales
Source: Sci Rep. 2023 Dec 17;13:22431. doi: 10.1038/s41598-023-49822-7 (PMC10725480; doi:10.1038/s41598-023-49822-7)
Supplement: Supplementary file 1 — Supplementary Figures. [file 41598_2023_49822_MOESM1_ESM.pdf]

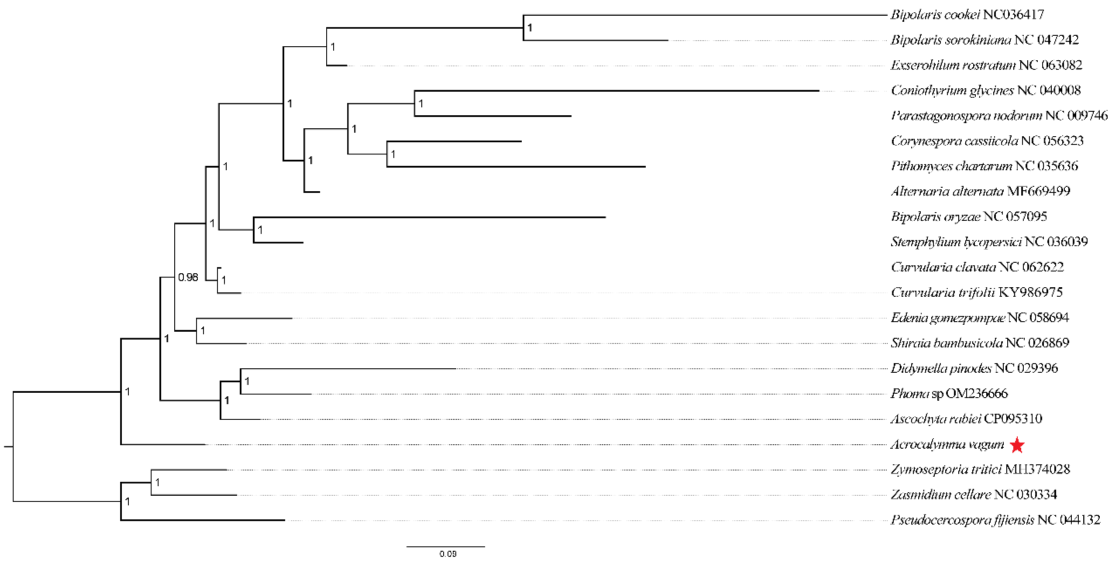

Figure S1 Phylogenetic trees of Pleosporales inferred by the MrBayes 3.2.6 methods based on amino acid sequences of 12 PCGs

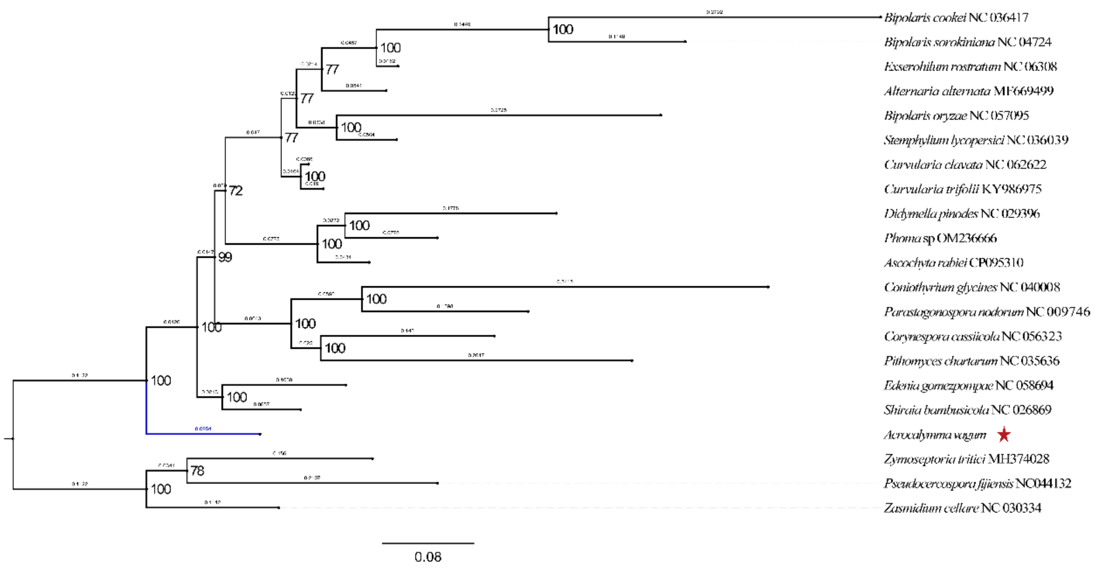

Figure S2 Phylogenetic relationships of Pleosporales inferred by maximum likelihood based on the nucleotides of the first and second codons of the 12 PCGs

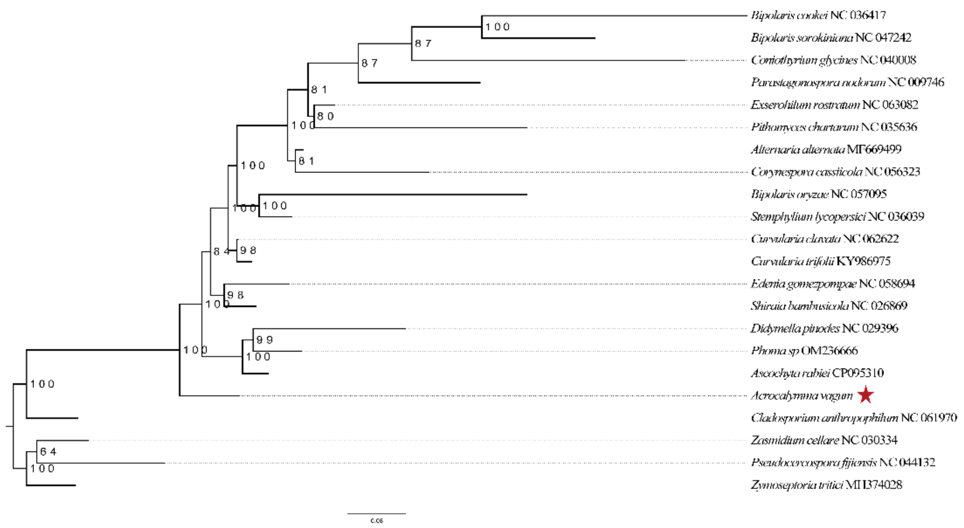

Figure S3 Phylogenetic trees of Pleosporales inferred by the maximum likelihood methods based on amino acid sequences of 12 PCGs
