# Supplementary material for: First complete mitogenome of Massarineae and its contribution to phylogenetic implications in Pleosporales
Source: Sci Rep. 2023 Dec 17;13:22431. doi: 10.1038/s41598-023-49822-7 (PMC10725480; doi:10.1038/s41598-023-49822-7)
Supplement: Supplementary file 2 — Supplementary Table S1. [file 41598_2023_49822_MOESM2_ESM.docx]

| Name of species | NCBI Reference Sequence | References |
| --- | --- | --- |
| *Bipolaris cookei* | NC 036417 | [25] |
| *Bipolaris sorokiniana* | NC 047242 | [26] |
| *Exserohilum rostratum* | NC 063082 | [27] |
| *Alternaria alternata* | MF 669499 | [28] |
| *Stemphylium lycopersici* | NC 036039 | [29] |
| *Curvularia clavata* | NC 062622 | Unpublished |
| *Curvularia trifolii* | KY 986975 | [30] |
| *Bipolaris oryzae* | NC 057095 | [31] |
| *Didymella pinodes* | NC 029396 | [32] |
| *Ascochyta rabiei* | CP 095310 | [33] |
| *Phoma* sp. | OM 236666 | [34] |
| *Corynespora cassiicola* | NC 056323 | [35] |
| *Parastagonospora nodorum* | NC 009746 | [36] |
| *Coniothyrium glycines* | NC 040008 | [37] |
| *Pithomyces chartarum* | NC 035636 | [38] |
| *Edenia gomezpompae* | NC 058694 | [39] |
| *Shiraia bambusicola* | NC 026869 | [40] |
| *Zymoseptoria tritici* | MH 374028 | [41] |
| *Pseudocercospora fijiensis* | NC 044132 | [42] |
| *Zasmidium cellare* | NC 030334 | [43] |

**Table S1:** NCBI numbers of the species used in the phylogenetic tree and references
