# Supplementary material for: First complete mitogenome of Massarineae and its contribution to phylogenetic implications in Pleosporales
Source: Sci Rep. 2023 Dec 17;13:22431. doi: 10.1038/s41598-023-49822-7 (PMC10725480; doi:10.1038/s41598-023-49822-7)
Supplement: Supplementary file 3 — Supplementary Table S2. [file 41598_2023_49822_MOESM3_ESM.docx]

**Table S2.** Gene annotation of the mitochondrial genome of *Acrocalymma vagum*

| Gene | Direction | Gene location | Size(bp) | Start Condon | Stop Condon | Anticodon | Intergenic nucleotides |
| --- | --- | --- | --- | --- | --- | --- | --- |
| *trnV* | N | 201-273 | 73 | - | - | GTA |  |
| *nad6* | N | 391-1395 | 1005 | ATG | TAA | - | 117 |
| *trnN* | N | 1618-1688 | 71 | - | - | AAC | 222 |
| *trnY* | N | 1778-1862 | 85 | - | - | TAC | 89 |
| *rrnS* | N | 1910-3701 | 1792 | - | - |  | 47 |
| *trnR* | N | 3758-3828 | 71 | - | - | AGA | 56 |
| *COB* | J | 5015-6184 | 1170 | ATG | TAG | - | 51 |
| *TrnV* | J | 6265-6337 | 73 | - | - | GTA | 80 |
| *TrnK* | J | 6548-6619 | 72 | - | - | AAA | 210 |
| *trnG* | J | 6631-6702 | 72 | - | - | GGA | 11 |
| *trnD* | J | 6705-6777 | 73 | - | - | GAC | 2 |
| *trnS1* | J | 6871-6950 | 80 | - | - | AGC | 93 |
| *trnW* | J | 7134-7205 | 72 | - | - | TGA | 183 |
| *trnI* | J | 7347-7418 | 72 | - | - | ATC | 141 |
| *trnR* | J | 7423-7493 | 71 | - | - | CGT | 4 |
| *trnS2* | J | 7529-7613 | 85 | - | - | TCA | 35 |
| *trnP* | J | 7741-7813 | 73 | - | - | CCA | 127 |
| *rrnL* | J | 7871-11363 | 3493 | - | - | - | 57 |
| *trnT* | J | 11360-11430 | 71 | - | - | ACA | -4 |
| *trnM* | J | 11453-11523 | 71 | - | - | ATG | 22 |
| *trnM* | J | 11529-11601 | 73 | - | - | ATG | 5 |
| *trnL2* | J | 11907-11989 | 83 | - | - | TTA | 306 |
| *trnE* | J | 12086-12158 | 73 | - | - | GAA | 96 |
| *trnA* | J | 12182-12253 | 72 | - | - | GCA | 23 |
| *trnF* | J | 12326-12399 | 74 | - | - | TTC | 72 |
| *trnL1* | J | 12519-12601 | 83 | - | - | CTA | 119 |
| *trnQ* | J | 12808-12879 | 72 | - | - | CAA | 206 |
| *trnH* | J | 12893-12965 | 73 | - | - | CAC | 13 |
| *trnM* | J | 13252-13323 | 72 | - | - | ATG | 286 |
| *nad4* | J | 13760-16645 | 2886 | TTA | TAA | - | 436 |
| *nad4l* | J | 17409-17678 | 270 | ATG | TAA | - | 763 |
| *nad5* | J | 17675-20122 | 2448 | ATA | TAA | - | -4 |
| *trnV* | J | 20207-20279 | 73 | - | - | GTA | 84 |
| *nad1* | J | 20496-21671 | 1176 | GTG | TAA | - | 216 |
| *cox2* | N | 25237-25989 | 753 | CTT | TAG | - | 3565 |
| *cox1* | N | 25979-27717 | 1739 | ATA | T-- | - | -21 |
| *trnC* | N | 28078-28147 | 70 | - | - | TGC | 360 |
| *nad3* | N | 28927-29886 | 960 | ATG | TAA | - | 764 |
| *nad2* | N | 29887-31638 | 1752 | ATT | TAA | - | -2 |
| *cox3* | N | 32523-33332 | 810 | ATG | TAA | - | 884 |
| *atp6* | N | 34050-34820 | 771 | ATG | TAA | - | 717 |
